# Left Coronary Artery to Pulmonary Trunk Fistula: Two Case Reports With Literature Review

**DOI:** 10.7759/cureus.43672

**Published:** 2023-08-17

**Authors:** Aamir Saeed, Ghulam Mujtaba Ghumman, Danial Mir, Ali Akram Khan, Kotikalapudi Sivarama

**Affiliations:** 1 Internal Medicine, Merit Health Wesley Hospital, Hattiesburg, USA; 2 Internal Medicine, St. Vincent Mercy Medical Center, Toledo, USA; 3 Medicine, Merit Health Wesley, Hattiesburg, USA; 4 Medicine, Icahn School of Medicine at Mount Sinai/Queens Hospital Center, New York City, USA; 5 Cardiology, McLaren Flint, Flint, USA

**Keywords:** coronary steal phenomenon, left heart cardiac catheterization, multiple detector computed tomography, coronary artery fistul, pulmonary trunk

## Abstract

Coronary artery fistulas (CAFs) are abnormal communication between coronary arteries and the pulmonary trunk or with adjacent heart structures. Coronary pulmonary artery fistulas (CPAFs) can be congenital or acquired. Mostly, CAFs are found as incidental findings on angiographic evaluation. The management of CPAFs varies from case to case depending on size, anatomical location, patient's clinical presentation, and presence of coronary steal phenomenon. We present two cases of CPAFs; one of them had coronary steal phenomena at a young age with no past medical history of coronary artery disease, and the patient underwent transcatheter coil embolization to close the fistula. In other cases, a fistulous connection between the left anterior descending (LAD) and the pulmonary trunk was found incidentally on computed tomography (CT) of the heart and based on a small-sized fistula and symptomatic improvement, the patient was discharged with conservative management. CPAFs are rare cardiac anomalies but can give rise to severe hemodynamic complications, so this should be a part of the initial differential diagnosis if the patient does not have significant coronary artery disease. Percutaneous closure or surgical correction is indicated if the patients are symptomatic or have secondary complications.

## Introduction

Coronary artery fistulas (CAFs) are direct communication of coronary arteries with the pulmonary trunk and adjacent heart structures, including cardiac chambers and the vessels [1]. The fistulas can be congenital or acquired in etiology. The acquired types can result from trauma, including stab wound injuries or iatrogenic from coronary artery interventions [1]. The incidence of CAFs is 0.002% in the general population and is diagnosed in around 0.25% of the individuals undergoing cardiac catheterization. They account for approximately 14% of all congenital cardiac defects; however, coronary-pulmonary artery fistula (CPAF) is found in 17% of the cases of CAFs [2]. Most of these patients can be asymptomatic (incidental finding on coronary angiography), while symptomatic patients present with chest pain, heart failure, or arrhythmias [3]. Coronary angiography can define the origin of the fistula, the number of fistulas, and the presence of an aneurysm. However, due to its limitations with the course of the fistula and anatomical relationships, multidetector computed tomography (MDCT) is routinely used to evaluate CPAFs in suspected patients along with coronary angiography [4]. We present two cases of left anterior descending (LAD) to pulmonary artery fistula diagnosed on coronary angiography. 

## Case presentation

Case 1

**Figure 1:**
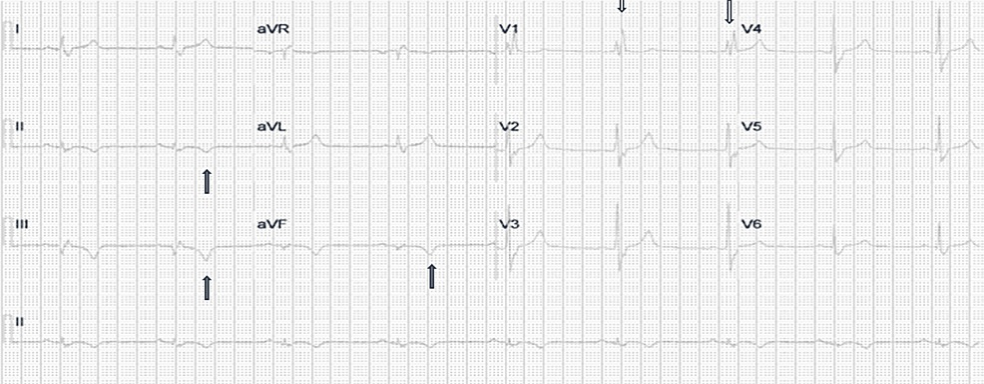
12-Lead EKG showing sinus bradycardia, right bundle branch pattern (white arrows), and T-wave inversions in inferior leads.

A 38-year-old male with no significant past medical history presented to the emergency department (ED) with complaints of substernal chest pain for the past several weeks. His pain was exertional, pressure-like, 7/10 in intensity, and non-radiating. The pain was not associated with nausea, vomiting, palpitations, or significant dyspnea. These episodes usually lasted from 10-15 minutes and were relieved on rest. He also reported having one episode of syncope. His pain worsened; therefore, he decided to come to the ED. Vital signs showed a heart rate of 55 beats/min, blood pressure of 110/70 mmHg, respiratory rate of 20 breaths/min, and oxygen saturation (SpO_2_) of 98% on room air. His cardiopulmonary examination was unremarkable. Initial EKG showed sinus bradycardia with right bundle branch block and T-wave inversion in the inferior leads (Figure 1).

**Figure 2:**
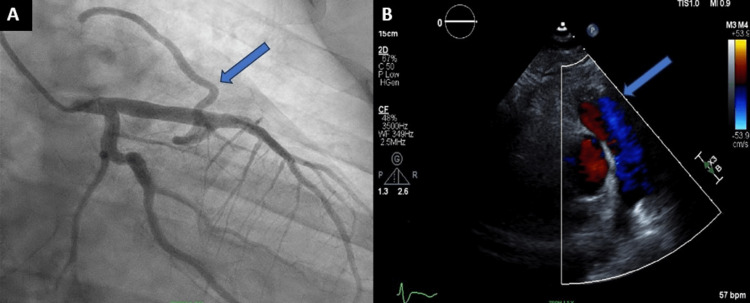
(A) Cardiac catheterization image showing mid-LAD to pulmonary artery fistula. (B) Doppler echocardiogram image showing the shunting. LAD - left anterior descending

Initial troponin was 3.56ng/mL. A computerized tomography (CT) pulmonary angiography ruled out pulmonary embolism. The patient was loaded with aspirin and started on intravenous (IV) heparin and nitroglycerin. Left heart catheterization (LHC) showed proximal right coronary artery (RCA) chronic total occlusion with satisfactory collaterals from the left side (LAD). The left coronary circulation did not have any significant disease. It also revealed a fistula between the LAD artery and pulmonary artery leading to suspicion of coronary steal syndrome. Transthoracic echocardiogram showed a normal ejection fraction of 50%-55% with no regional wall motion abnormalities. The bubble study was positive for a left-to-right shunt. Color Doppler on echo showed a communicating fistula into the pulmonary artery (Figures 2A, 2B).

His pain improved with the addition of isosorbide mononitrate. His troponin peaked at 5.46ng/mL (normal range 0-0.04ng/mL) and then started trending down. Non-ST elevation myocardial infarction (NSTEMI) was thought to occur from coronary steal secondary to the communicating fistula between the LAD and the pulmonary artery. He was discharged in stable condition once he remained chest pain-free for 24 hours.

One week after discharge, the patient presented to the ED with similar complaints of chest pain, and EKG was unchanged from the previous visit. The patient was hemodynamically stable, and his examination was unremarkable. Troponin on arrival was 0.205ng/mL, which was lower than the one he had prior to discharge. Considering that the patient was continuing to have symptoms of ischemia, the patient underwent transcatheter coil embolization to close the fistula. Symptoms improved after the closure of the fistula. The patient remained asymptomatic on a two-week follow-up and to date.

Case 2 

**Figure 3:**
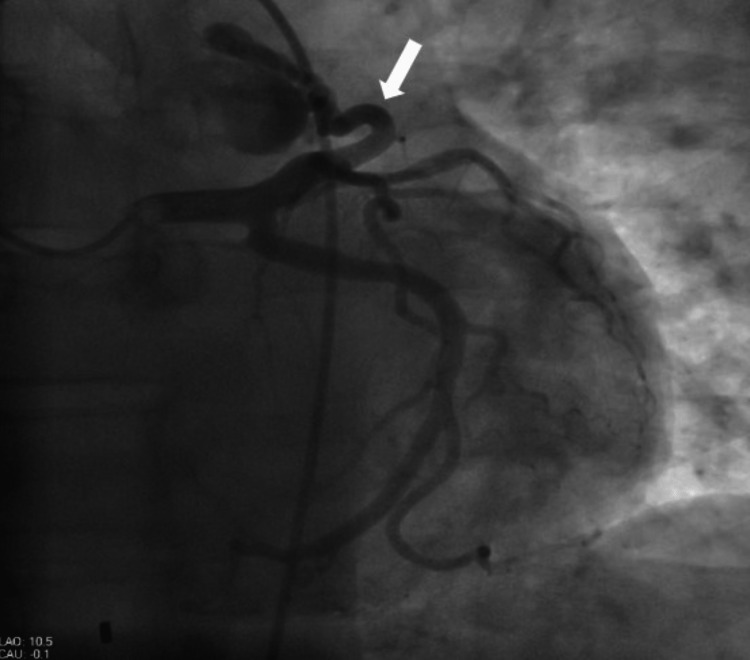
Selected cardiac catheterization image showing a fistula between the left anterior descending artery and pulmonary artery.

A 62-year-old male with a history of hypertension, gastroesophageal reflux disease, and type 2 diabetes mellitus presented to the ED with dull pressure-like chest pain on exertion. He denied any palpitations, dyspnea, dizziness, and syncopal attacks. Vital signs were stable, and general physical examination, including cardiopulmonary examination, was unremarkable. EKG revealed normal sinus rhythm with no acute ST segment changes. Serial troponins were unremarkable. Transthoracic echocardiography showed normal left ventricular systolic function with an ejection fraction (EF) of 55%-60% with no valvular abnormality. Single photon emission computerized tomography (SPECT) myocardial perfusion imaging study showed a low probability for myocardial ischemia and EF 53% at rest and 56% at stress, with no EKG changes on the rest and stress. We also performed computed tomography (CT) of the heart for calcium scoring, which was 4 (calcium score <100 is low risk) with minimal evidence of coronary artery disease (CAD). CT coronary angiogram with IV contrast for anatomic relationships showed a fistulous connection between LAD and pulmonary trunk. An LHC revealed nonobstructive CAD, 60% left ventricular ejection fraction, and a small fistula between mid-LAD artery and the pulmonary artery (Figure 3).

Anginal symptoms improved with Ranolazine. Based on improvement in symptoms, and small fistula size, the decision was made for no intervention. The patient remained asymptomatic on four weeks of follow-up and to date.

## Discussion

Coronary-pulmonary artery fistula (CPAF) is a rare subtype of CAF with prevalence rates ranging from 0.32% to 0.68% [5]. The common draining sites for CAFs are the right atrium, right ventricle, pulmonary artery, and coronary sinus, leading to left to right cardiac shunting. In our cases, both patients had LAD fistula with the pulmonary artery. Most CPAFs are congenital but can be acquired, cardiac disease-related, traumatic, or iatrogenic. Acquired causes include acute myocardial infarction, hypertrophic cardiomyopathy, dilated cardiomyopathy, and malignancy, while iatrogenic causes include percutaneous coronary intervention, coronary artery bypass grafting, cardiac transplant, endomyocardial biopsy, and permanent pacemaker placement [6].

The clinical manifestations of individuals with CPAFs varied from asymptomatic to symptomatic based on the origin, size, number of fistulas, left to right shunting, and coronary steal phenomenon. Most of the asymptomatic patients with CPAFs have small fistulas with insignificant shunting of the blood [7]. CAFs are usually asymptomatic during the first two decades of life. After that, the frequency of symptoms and complications increased with age [8]. The most common presenting symptom is angina due to CAD and coronary steal phenomenon in the absence of CAD. The primary pathophysiological mechanism behind ischemia in symptomatic patients with CPAFs is coronary steal syndrome, which is the shunting of the blood to the low-resistance receiving cavity (e.g., pulmonary vessels) and leaving behind ischemia in the areas with high-resistance to the blood flow [9]. It is hypothesized that with the absence of atherosclerotic disease in the coronary arteries, the steal phenomenon is the cause of the ischemic symptoms, which is evident by the increase in the myocardial oxygen demand in strenuous activity. Other complications of CPAFs include congestive heart failure, fistula rupture, endocarditis, thrombosis, embolism, pulmonary hypertension, and arrhythmias [8]. Fistulas can be significant (>250mm) and dilated or tortuous and tend to enlarge over time. The fistulas can rupture spontaneously and can cause hemopericardium, cardiac tamponade, and sudden cardiac death [10].

EKG, echocardiogram, cardiac stress test, MDCT, and cardiac magnetic resonance imaging are the preferred non-invasive investigations in symptomatic patients with suspected CPAFs. Coronary angiography is the gold standard diagnostic technique for diagnosing the fistula, which provides detailed anatomic information about the size, origin, course, and drainage of the fistulas and associated CAD [11]. However, in some patients, the source and anatomical relation of the CPAFs to the adjacent structures is difficult to determine; in such cases, imaging tools are used as adjuncts. MDCT provides an excellent three-dimensional reconstruction of the fistula, its origin, size, stenosis, aneurysm, and relation with adjacent cardiac structures [4]. Any obstruction along the course can be determined with MDCT. A myocardial perfusion scan is a valuable tool in patients with small-size CPAFs, and it can help patients who need invasive treatment or conservative pharmacotherapy. Myocardial perfusion positron emission tomography (PET) is an advanced technology with better spatial resolution and sensitivity than SPECT. If the patient has limited myocardial ischemia, it can be treated conservatively [7,4,9,11].

There are no established guidelines for managing CPAFs; however, the current regime varies based on the presence or absence of symptoms. The American College of Cardiology/American Heart Association 2018 guidelines for managing adults with congenital heart disease recommend the closure of all sizeable CAFs regardless of the symptoms [12]. Similarly, closure is indicated in symptomatic patients with small-size fistulas. Asymptomatic patients can be treated conservatively with follow-up echocardiography in three to five years to monitor the size of the fistulas over time [8,10,11]. Treatment options are medical management, surgical ligation, and transcatheter coil embolization. Improvements in symptoms have been reported with antianginal medications in patients with small CPAFs with no significant shunting. In our second case, the patient was managed with anti-anginal medications considering small size fistula, and the patient's symptoms improved with medical therapy.

No comparison studies have been done throughout the literature between transcatheter closure and surgical correction. However, transcatheter closure has become the treatment of choice in patients with proximal fistula origin, single drainage site, and no other cardiac disease [12]. Surgical ligation is needed in patients with multiple drainage sites, distal fistula origin, tortuosity of the vessels, significant aneurysms, pulmonary-systemic flow ratio exceeding 1.5:1, and associated cardiac diseases [13]. Patients with CPAFs who undergo closure have an excellent prognosis; recurrence rates are as low as 9%-19% in transcatheter closure and 25% with surgical ligation. The prognosis depends upon the shunt's severity, associated complications, pulmonary hypertension, heart failure, and bacterial endocarditis [12,13]. Our first case was managed with transcatheter coil embolization leading to the resolution of the patient's symptoms. Transcatheter closure is less invasive and less expensive, associated with low morbidity and mortality, and less recovery time than surgical management.

## Conclusions

CPAFs are rare types of CAFs. Most patients are asymptomatic and do not require specific treatment; however, the potential for life-threatening complications, arrhythmias, myocardial infarction, and sudden cardiac death are relevant in young patients and athletes and require correction. Symptomatic patients usually present with recurrent angina or arrhythmias. Due to the low prevalence of this anomaly, there are currently no guidelines to treat symptomatic vs. asymptomatic CPAFs. In this case series, we described two cases of direct communication of the LAD artery to the pulmonary artery. We believe this case series will contribute to a paradigm shift, where the coronary arteries' assessment should not only be done with suspicion of atherosclerotic disease of the coronary arteries but also with consideration of the fistulas, especially in younger patients with recurrent nature of symptoms.
